# Draft annotated genome of the entomopathogenic fungus *Metarhizium pingshaense* isolated from *Spodoptera* species

**DOI:** 10.1128/mra.00410-24

**Published:** 2024-12-23

**Authors:** Nonthakorn (Beatrice) Apirajkamol, Andreas Bachler, Wee Tek Tay, Bishwo Mainali, Phillip Taylor, Thomas Kieran Walsh

**Affiliations:** 1 Applied BioSciences, Macquarie University, Sydney, New South Wales, Australia; 2 Black Mountain Laboratories, Commonwealth Scientific and Industrial Research Organisation, Canberra, Australia; University of Maryland School of Medicine, Baltimore, Maryland, USA

**Keywords:** annotated genome, fungus, entomopathogenic fungus, *Metarhizium*, *Metarhizium pingshaense*, PacBio HiFi, whole genome sequencing

## Abstract

The entomopathogenic fungus *Metarhizium pingshaense* infects diverse insect host species. We present an annotated draft genome of *M. pingshaense* (Commonwealth Scientific and Industrial Research Organisation [CSIRO] strain M-1000) isolated from a *Spodoptera* species individual, thereby contributing to future research of *M. pingshaense* as a potential biological control agent.

## ANNOUNCEMENT

Although *Metarhizium pingshaense* is an entomopathogenic fungus to a wide range of insects including malaria-transmitting *Anopheles* mosquitoes (1, 2) and many lepidopteran species (3–6), *M. pingshaense* is considerably under-researched. The availability of a draft genome will advance research on this species to better explore its potential as a biological control agent against insect pests.

The *M. pingshaense* from Commonwealth Scientific and Industrial Research Organisation (CSIRO) fungal collection (M-1000; CSIRO Black Mountain, Canberra, Australia) was isolated from an infected *Spodoptera* species individual collected from the Cameron Highlands, Pahang State, Malaysia, in May 1993. *M. pingshaense* M-1000 spores had been freeze-dried and preserved at −80°C. The fungus was revived and cultured, and the spores were collected using previously published protocol (7).

The genomic DNA was extracted using the protocol of Apirajkamol et al. (8). The quality and quantity of the extracted DNA were analyzed using a NanoDrop and Qubit 2.0 fluorometer. Extracted DNA was submitted for library preparation and sequencing using PacBio HiFi Sequel ll sequencer with SMRTBell technology (Genomics WA; Perth, Australia). The DNA was fragmented with a Diagenode Megaruptor-3, size-selected using BluePippin, and assessed on a Femto-Pulse (>15 Kb). The library was prepared using the SMRTbell Prep Kit 3.0 (PN 102–166-600, PacBio). The number of raw reads is 366,149 with 73.71× coverage, and the read *N*_50_ is 9,668.

**Fig 1:**
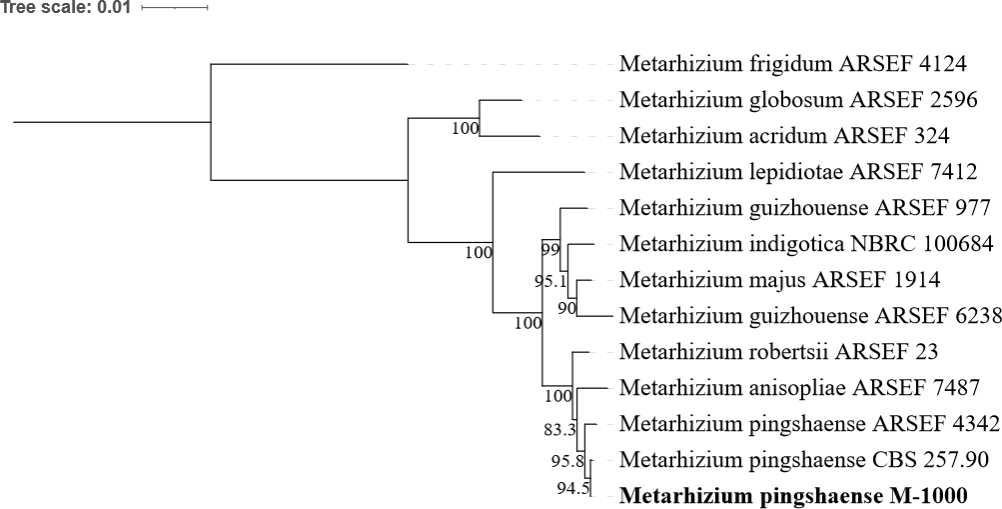
ML phylogenetic analysis of *M. pingshaense* CSIRO strain (M-1000, indicated in bold). The *M. pingshaense* M-1000 clustered with two other *M. pingshaense* accessions (ARSEF 4342, CBS 257.90) with high bootstrap node support estimates at 95.8% and 94.5%. Refer to the main text for analysis methods.

We confirmed the species of the *M. pingshaense* M-1000 strain through phylogenetic analysis using seven DNA markers including DNA lyase (APN2), beta-tubulin, RNA polymerase II largest subunit (RPB1)a, RPB1b, RNA polymerase II second largest subunit (RPB2)a, RPB2b, translation elongation factor (9). The sequences were aligned using MUSCLE with default parameters (Muscle 5.1 with algorithm: PPP, Geneious Prime 2023.2.1) (10). A maximum likelihood (ML) phylogeny was constructed using IQ-tree <http://iqtree.cibiv.univie.ac.at/> (11) specifying auto-selection for the best fit evolutionary substitution model, and with 1,000 bootstrap replications to estimate branch node confidence values. The phylogeny was edited via the iTOL v.6 webserver (https://itol.embl.de/) (12). The results supported the identification of M-1000 as *M. pingshaense*, with M-1000 clustering confidently at 94.5% with the known *M. pingshaense* CBC257.90 accession (Fig. 1).

The *M. pingshaense* genome was *de novo* assembled by Canu 2.1.1 (13) with default parameters. The quality and completeness of the assembled genome were determined using QUAST 5.2.0 (14) and BUSCO 5.2.2 (15), with hypocreales_odb10 (the order of M-1000) data set with default parameters. The genome size of *M. pingshaense* (M-1000) was estimated to be 44,398,422 bp with 49.87% GC with 97% complete BUSCOs (Table 1). Five misassembled mitochondrial gene contigs were manually removed prior to the genome being uploaded to GenBank.

**TABLE 1:** Summary of *M. pingshaense* M-1000 genome assembly and annotation

Parameter	Genome assembly	Genome annotation
Contigs	121	
Largest contig	7,626,275	
N50	4,567,404	
N90	2,344,541	
L50	5	
L90	9	
Total length (bp)	44,398,422	
GC content (%)	49.87	
No. expected chromosome	8	
Complete BUSCOs (%)	97 (4361)	96.2 (4322)
Complete and single-copy BUSCOs (%)	94.2 (4233)	93.5 (4200)
Complete and duplicated BUSCOs (%)	2.8 (128)	2.7 (122)
Fragmented BUSCOs (%)	0.6 (27)	1.6 (74)
Missing BUSCOs (%)	2.4 (106)	2.2 (98)
Total BUSCO groups searched	4,494	4,494
No. of predicted genes		12,150

The genome was structurally annotated with Augustus (16) through Galaxy Australia (Galaxy Version 3.4.0+galaxy1 https://usegalaxy.org.au/) with default settings (17) using the *Fusarium graminearum* genome as reference. The annotation was converted to a standard gff3 format using the command line “agat_convert_sp_gxf2gxf.pl” (18). The number of predicted genes from the draft genome is 12,150, and the complete BUSCOs for the predicted proteins from the annotated genome are 96.2% (see Table 1).

## Data Availability

The genome is available at NCBI for both the assembly (ASM4137997v1) and raw reads (SRR28535458). The annotation is available in (19).
